# Supplementary material for: Barriers and facilitators to perioperative smoking cessation: A scoping review
Source: PLoS One. 2024 Jun 11;19(6):e0298233. doi: 10.1371/journal.pone.0298233 (PMC11166293; doi:10.1371/journal.pone.0298233)
Supplement: S1 Fig — (DOCX) [file pone.0298233.s001.docx]

**S1 Fig**. Number of barriers and facilitators mapped to each TDF domain.
