# Supplementary material for: Barriers and facilitators to perioperative smoking cessation: A scoping review
Source: PLoS One. 2024 Jun 11;19(6):e0298233. doi: 10.1371/journal.pone.0298233 (PMC11166293; doi:10.1371/journal.pone.0298233)
Supplement: S2 Table — (DOCX) [file pone.0298233.s003.docx]

**S2 Table. Search Strategies**

**MEDLINE** (Searched August 11, 2022) Search Strategy:

| **#** | **Searches** | **Results** |
| --- | --- | --- |
| 1 | smoking cessation*.mp. or exp Smoking Cessation/ | 43355 |
| 2 | smoking.mp. or exp Smoking/ | 316502 |
| 3 | exp Cigarette Smoking/ or cigarette*.mp. | 81408 |
| 4 | exp "Tobacco Use"/ or exp Tobacco/ or exp "Tobacco Use Cessation"/ or exp "Tobacco Use Disorder"/ or tobacco.mp. or exp Tobacco Smoking/ | 146157 |
| 5 | smoker*.mp. or exp Smokers/ | 97650 |
| 6 | or/1-5 | 415255 |
| 7 | surgery.mp. or exp General Surgery/ | 2936492 |
| 8 | (preoperative or pre-operative).mp. or exp Preoperative Care/ or exp Preoperative Period/ | 361088 |
| 9 | (postoperative or post-operative).mp. or exp Postoperative Care/ or exp Postoperative Period/ | 934355 |
| 10 | (perioperative or peri-operative).mp. or exp Perioperative Care/ or exp Perioperative Period/ | 342884 |
| 11 | (presurgical or pre-surgical).mp. | 13102 |
| 12 | (postsurgical or post-surgical).mp. | 25979 |
| 13 | (perisurgical or peri-surgical).mp. | 202 |
| 14 | or/7-13 | 3249187 |
| 15 | (barrier* or facilitat* or challenge* or success* or constrain* or difficult* or interfer* or obstruct* or problem* or restrain* or restrict* or enabler* or factor* or determinant* or variable* or covariable* or predictor* or attitude* or practice* or perception*).mp. | 12621445 |
| 16 | anesthesiologist*.mp. or exp Anesthesiologists/ | 26106 |
| 17 | anesthesia.mp. or exp Anesthesia/ | 298783 |
| 18 | anesthetist*.mp. or exp Anesthetists/ | 8336 |
| 19 | exp Surgeons/ or surgeon*.mp. | 238196 |
| 20 | nurse*.mp. | 392741 |
| 21 | (physician or doctor or clinician).mp. or exp Physicians/ | 506176 |
| 22 | or/16-21 | 1367247 |
| 23 | exp Animals/ not exp Humans/ | 5035392 |
| 24 | (6 and 14 and 15 and 22) not 23 | 3230 |
| 25 | limit 24 to yr="2000 -Current" | 2896 |

**Embase** (Searched August 11, 2022) Search Strategy:

| **#** | **Searches** | **Results** |
| --- | --- | --- |
| 1 | exp smoking cessation program/ or exp smoking cessation/ or smoking cessation*.mp. | 73302 |
| 2 | exp smoking/ or smoking.mp. | 550636 |
| 3 | cigarette*.mp. or exp cigarette smoking/ | 134065 |
| 4 | exp tobacco/ or exp "tobacco use"/ or exp tobacco consumption/ or tobacco.mp. or exp tobacco dependence/ | 536698 |
| 5 | smoker*.mp. or exp current smoker/ | 156222 |
| 6 | or/1-5 | 663379 |
| 7 | surgery.mp. or exp surgery/ | 6154883 |
| 8 | exp preoperative treatment/ or exp preoperative care/ or exp preoperative period/ or preoperative.mp. or pre-operative.mp. | 648335 |
| 9 | exp postoperative period/ or exp postoperative care/ or postoperative.mp. or post-operative.mp. | 1424336 |
| 10 | exp perioperative period/ or perioperative.mp. or peri-operative.mp. | 201314 |
| 11 | (presurgical or pre-surgical).mp. | 20098 |
| 12 | (postsurgical or post-surgical).mp. | 39019 |
| 13 | (perisurgical or peri-surgical).mp. | 338 |
| 14 | or/7-13 | 6274362 |
| 15 | (barrier* or facilitat* or attitude* or practice* or perception*).mp. | 3882412 |
| 16 | exp anesthesiology/ or exp anesthesiologist/ or anesthesiologist*.mp. | 59927 |
| 17 | anesthesia.mp. or exp anesthesia/ | 441459 |
| 18 | anesthetist*.mp. or exp anesthesist/ | 41588 |
| 19 | exp surgeon/ or surgeon*.mp. | 415941 |
| 20 | exp nurse/ or nurse*.mp. | 463311 |
| 21 | exp physician/ or (physician or doctor or clinician).mp. | 1300115 |
| 22 | or/16-21 | 2320758 |
| 23 | exp animal/ not exp human/ | 4982414 |
| 24 | (6 and 14 and 15 and 22) not 23 | 2677 |
| 25 | limit 24 to yr="2000-Current" | 2585 |

**CENTRAL** (Searched August 11, 2022)

| **ID** | **Search** | **Hits** |
| --- | --- | --- |
| #1 | MeSH descriptor: [Smoking] explode all trees | 6522 |
| #2 | MeSH descriptor: [Smoking Cessation] explode all trees | 4455 |
| #3 | MeSH descriptor: [Tobacco] explode all trees | 204 |
| #4 | MeSH descriptor: [Tobacco Use Cessation] explode all trees | 128 |
| #5 | (smoking cessation* or smoking or cigarette* or tobacco or smoker*) | 44667 |
| #6 | {OR #1-#5} | 44667 |
| #7 | MeSH descriptor: [General Surgery] explode all trees | 366 |
| #8 | MeSH descriptor: [Perioperative Care] explode all trees | 12808 |
| #9 | MeSH descriptor: [Preoperative Care] explode all trees | 6150 |
| #10 | MeSH descriptor: [Perioperative Period] explode all trees | 9262 |
| #11 | (preoperative or postoperative or perioperative or presurgical or postsurgical or perisurgical) | 159238 |
| #12 | {OR #7-#11} | 162584 |
| #13 | (barrier* or facilitat* or challenge* or success* or constrain* or difficult* or interfer* or obstruct* or problem* or restrain* or restrict* or enabler* or factor* or determinant* or variable* or covariable* or predictor* or attitude* or practice* or perception*) | 710177 |
| #14 | MeSH descriptor: [Physicians] explode all trees | 2430 |
| #15 | MeSH descriptor: [Nurses] explode all trees | 1323 |
| #16 | MeSH descriptor: [Anesthetists] explode all trees | 125 |
| #17 | (physician* or doctor* or clinician* or nurse* or anesthetist*) | 122085 |
| #18 | {OR #14-#17} | 122515 |
| #19 | #6 AND #12 AND #13 AND #18 with Cochrane Library publication date from Jan 2000 to present | 363 |

| **CINAHL (August 11, 2022)** | | | | |
| --- | --- | --- | --- | --- |
| **#** | **Query** | **Limiters/Expanders** | **Last Run Via** | **Results** |
| S5 | S1 AND S2 AND S3 AND S4 | Limiters - Published Date: 20000101-20221231 Expanders - Apply equivalent subjects Search modes - Boolean/Phrase | Interface - EBSCOhost Research Databases Search Screen - Advanced Search Database - CINAHL | 1,035 |
| S4 | (MH "Physicians+") OR (MH "Nurse Anesthetists") OR (MH "Anesthetists+") OR (MH "Surgeons") OR ""anesthetist* OR surgeon* OR nurse* OR physician* OR doctor* OR clinician*"" | Expanders - Apply equivalent subjects Search modes - Boolean/Phrase | Interface - EBSCOhost Research Databases Search Screen - Advanced Search Database - CINAHL | 1,015,726 |
| S3 | ""barrier* OR facilitat* OR challenge* OR success* OR constrain* OR difficult* OR interfer* OR obstruct* OR problem* OR restrain* OR restrict* OR enabler* OR factor* OR determinant* OR variable* OR covariable* OR predictor* OR attitude* OR practice* OR perception*"" | Expanders - Apply equivalent subjects Search modes - Boolean/Phrase | Interface - EBSCOhost Research Databases Search Screen - Advanced Search Database - CINAHL | 3,362,120 |
| S2 | (MH "Postoperative Period") OR (MH "Preoperative Period+") OR (MH "Preoperative Care+") OR (MH "Postoperative Care+") OR ""surgery OR preoperative OR postoperative OR perioperative OR presurgical or postsurgical OR perisurgical"" | Expanders - Apply equivalent subjects Search modes - Boolean/Phrase | Interface - EBSCOhost Research Databases Search Screen - Advanced Search Database - CINAHL | 712,179 |
| S1 | (MH "Smoking Cessation") OR (MH "Smoking Cessation Programs") OR (MH "Smoking+") OR (MH "Tobacco Products+") OR (MH "Tobacco Use Cessation Products+") OR ""smoking cessation* OR smoking OR cigarette* OR tobacco OR smoker*"" | Expanders - Apply equivalent subjects Search modes - Boolean/Phrase | Interface - EBSCOhost Research Databases Search Screen - Basic Search Database - CINAHL | 142,270 |

**PsycINFO** (Searched August 11, 2022) Search Strategy:

| **#** | **Searches** | **Results** |
| --- | --- | --- |
| 1 | exp Smoking Cessation/ or smoking cessation*.mp. | 19613 |
| 2 | smoking.mp. | 64866 |
| 3 | cigarette*.mp. | 24069 |
| 4 | exp Tobacco Smoking/ or tobacco.mp. or exp "Tobacco Use Disorder"/ | 48122 |
| 5 | smoker*.mp. | 24341 |
| 6 | or/1-5 | 76089 |
| 7 | surgery.mp. or exp Surgery/ | 93213 |
| 8 | (preoperative or pre-operative).mp. | 5391 |
| 9 | (postoperative or post-operative).mp. | 12550 |
| 10 | (perioperative or peri-operative).mp. | 1546 |
| 11 | (presurgical or pre-surgical).mp. | 1957 |
| 12 | (postsurgical or post-surgical).mp. | 2955 |
| 13 | (perisurgical or peri-surgical).mp. | 13 |
| 14 | or/7-13 | 98517 |
| 15 | (barrier* or facilitat* or challenge* or success* or constrain* or difficult* or interfer* or obstruct* or problem* or restrain* or restrict* or enabler* or factor* or determinant* or variable* or covariable* or predictor* or attitude* or practice* or perception*).mp. | 3221227 |
| 16 | exp Anesthesiology/ or anesthesiologist*.mp. | 844 |
| 17 | anesthesia.mp. | 7400 |
| 18 | anesthetist*.mp. | 291 |
| 19 | exp Surgeons/ or surgeon*.mp. | 4463 |
| 20 | nurse*.mp. or exp Nurses/ | 81761 |
| 21 | exp Physicians/ or (physician* or doctor* or clinician*).mp. | 235496 |
| 22 | or/16-21 | 310749 |
| 23 | 6 and 14 and 15 and 22 | 97 |
| 24 | limit 23 to yr="2000-Current" | 83 |
