# Supplementary material for: Barriers and facilitators to perioperative smoking cessation: A scoping review
Source: PLoS One. 2024 Jun 11;19(6):e0298233. doi: 10.1371/journal.pone.0298233 (PMC11166293; doi:10.1371/journal.pone.0298233)
Supplement: S3 Table — (DOCX) [file pone.0298233.s004.docx]

##### ***S3 Table*.** Quality assessment of included studies with the Mixed Methods Appraisal Tool (MMAT)

| **Randomized controlled trials** | | | | | |  |
| --- | --- | --- | --- | --- | --- | --- |
| Author | *2.1. Is randomization appropriately performed?* | *2.2. Are the groups comparable at baseline?* | *2.3. Are there complete outcome data?* | *2.4. Are outcome assessors blinded to the intervention provided?* | *2.5 Did the participants adhere to the assigned intervention?* | *Overall rating* |
| Newhall 2017 | Yes | Yes | Yes | No | Yes | 80% |
| Rajaee 2019 | Yes | Yes | Yes | No | Yes | 80% |

| **Qualitative studies** | | | | | |  |
| --- | --- | --- | --- | --- | --- | --- |
| Author, year | *1.1. Is the qualitative approach appropriate to answer the research question?* | *1.2. Are the qualitative data collection methods adequate to address the research question?* | *1.3. Are the findings adequately derived from the data?* | *1.4. Is the interpretation of results sufficiently substantiated by data?* | *1.5. Is there coherence between data sources, collection, analysis and interpretation?* | *Overall rating* |
| Farley 2016 | Yes | Yes | Yes | Yes | Yes | 100% |
| Luxton 2019 | Yes | Yes | Yes | Yes | Yes | 100% |
| Newhall 2016 | Yes | Yes | Yes | Yes | Yes | 100% |
| Warner 2008 | Yes | Yes | Yes | Yes | Yes | 100% |

| **MMAT – Mixed-methods studies** | | | | | |  |
| --- | --- | --- | --- | --- | --- | --- |
| Author | 5.1. Is there an adequate rationale for using a mixed method design to address the research question? | 5.2. Are the different components of the study effectively integrated to answer the research question? | 5.3. Are the outputs of the integration of qualitative and quantitative components adequately interpreted? | 5.4. Are divergences and inconsistencies between quantitative and qualitative results adequately addressed? | 5.5. Do the different components of the study adhere to the quality criteria of each tradition of the methods involved? | Overall rating |
| Bottorff 2016 | Yes | Yes | Yes | Yes | No | 80% |
| Jose 2020 | Yes | Yes | Yes | Yes | Yes | 100% |
| McDonnell 2014 | Yes | Yes | Yes | Yes | Yes | 100% |
| McDonnell 2016 | Yes | Yes | Yes | Yes | Yes | 100% |
| Rosvall 2017 | Yes | Yes | Yes | Yes | Yes | 100% |

| **Cross-sectional surveys** | | | | | |  |
| --- | --- | --- | --- | --- | --- | --- |
| Author | *4.1. Is the sampling strategy relevant to address the research question?* | *4.2. Is the sample representative of the target population?* | *4.3. Are the measurements appropriate?* | *4.4. Is the risk of nonresponse bias low?* | *4.5. Is the statistical analysis appropriate to answer the research question?* | *Overall rating* |
| Crews 2008 | Yes | Yes | Yes | No | Yes | 80% |
| Gay-Escoda 2012 | Yes | Yes | Yes | No | Yes | 80% |
| Houghton 2008 | Yes | Yes | Yes | No | Yes | 80% |
| Kai 2008 | Yes | Yes | Yes | No | Yes | 80% |
| Karabeyoglu 2014 | Yes | Yes | Yes | No | Yes | 80% |
| Marrufo 2019 | Yes | Yes | Yes | No | Yes | 80% |
| Owen 2007 | Yes | Yes | Yes | No | Yes | 80% |
| Saddichha 2010 | Yes | Yes | Yes | No | Yes | 80% |
| Schultz 2014 | Yes | Yes | Yes | No | Yes | 80% |
| Shannon-Cain 2002 | Yes | Yes | Yes | No | Yes | 80% |
| Shi 2010 | Yes | Yes | Yes | No | Yes | 80% |
| Smeds 2017 | Yes | Yes | Yes | Yes | Yes | 100% |
| Taniguchi 2011 | Yes | Yes | Yes | Yes | Yes | 100% |
| VanSlyke 2017 | Yes | Yes | Yes | No | Yes | 80% |
| Vick 2011 | Yes | Yes | Yes | No | Yes | 80% |
| Warner 2004 | Yes | Yes | Yes | No | Yes | 80% |
| Webb 2013 | Yes | Yes | Yes | No | Yes | 80% |
| Yankie 2006 | Yes | Yes | No | No | Yes | 60% |
| Yao 2009 | Yes | Yes | No | No | Yes | 60% |
| Yu 2013 | Yes | Yes | No | Yes | Yes | 80% |
