# Supplementary material for: Barriers and facilitators to perioperative smoking cessation: A scoping review
Source: PLoS One. 2024 Jun 11;19(6):e0298233. doi: 10.1371/journal.pone.0298233 (PMC11166293; doi:10.1371/journal.pone.0298233)
Supplement: S1 File — (DOCX) [file pone.0298233.s005.docx]

**S1 File: Reports excluded at full text screening stage**

**Abstract only (n=25):**

| 1. El-Hayani R, et al. Digital remote screening with proactive referral to improve smoking cessation referral rates in the head & neck clinic - a two-cycle audit. Oral Oncology. 2021;118(Supplement):15. |
| --- |
| 1. He W, et al. 752 Text Messages Targeting Smoking Cessation in Surgical Patients: Comparing Clinician and Consumer Review. Heart Lung and Circulation. 2020;29(Supplement 2):S375. |
| 1. Landefeld J, et al. Uncovering barriers to prescribing post-discharge nicotine replacement therapy for hospitalized smokers. Journal of General Internal Medicine. 2019;34(2 Supplement):S772. |
| 1. He W, et al. Development of Mobile Phone Text Messages Targeting Smoking Cessation in Surgical Patients. Heart Lung and Circulation. 2019;28(Supplement 4):S362-S363. |
| 1. Tsai Y, et al. Anaesthetists' and surgeons' attitude and behaviour towards smoking cessation-single centre study in Australia. Anaesthesia. 2018;73(Supplement 3):65. |
| 1. Sadek J, et al. A novel, evidence-based smoking cessation program in an outpatient colorectal surgery clinic: 1 Year outcomes. Diseases of the Colon and Rectum. 2018;61(5):e281. |
| 1. Seth B, et al. Provider-level barriers to adoption of a large-scale inpatient tobacco treatment service. Chest. 2017;152(4 Supplement 1):A1096. |
| 1. Ventola H, et al. Preoperative smoking cessation: Recording of smoking status in the Porvoo hospital area in Finland. Value in Health. 2017;20(9):A653. |
| 1. Nolan M, et al. Design, implementation and evaluation of a smoking cessation intervention for patients undergoing breast cancer surgery. Cancer Research. 2017;77(4 Supplement 1). |
| 1. Oswald N, et al. A national survey of thoracic surgery units: Pre-operative smoking cessation practice shows need for better provision of service. Lung Cancer. 2016;91(Supplement 1):S62. |
| 1. Kum F, et al. Surgeons & smoking: Discussing smoking cessation with patients in head & neck cancer clinics. European Journal of Surgical Oncology. 2016;42(9):S159. |
| 1. Ayyash R, et al. Surgical risk reduction-how far are patients willing to go?. Anaesthesia. 2016;71(Supplement 4):18. |
| 1. Patil A, et al. Introducing 'peri-operative smoking cessation' leaflet as a part of prehabilitation: A step at facilitating the role of anaesthetists as peri-operative physicians. Anaesthesia. 2016;71(Supplement 4):63. |
| 1. Sirivoranankul C, et al. The vape study: Veteran attitudes towards perioperative smoking cessation and E-Cigarette use. Anesthesia and Analgesia. 2016;122(5 Supplement 3):S457. |
| 1. Patil A, et al. Introducing 'peri-operative smoking cessation' leaflet as a part of pre-habilitation: A step at facilitating the role of anaesthetists as peri-operative physicians. Regional Anesthesia and Pain Medicine. 2016;41(5 Supplement 1):e156. |
| 1. Goodney P, et al. A pilot randomized trial of a brief smoking cessation intervention for patients with peripheral arterial disease: The vascular physicians offer and report (VAPOR) trial. Journal of Vascular Surgery. 2016;63(6 SUPPL. 1):204S. |
| 1. Fleetwood V, et al. Smoking policies among liver transplantation centers. American Journal of Transplantation. 2015;15(SUPPL. 1):101. |
| 1. Cheng L, et al. The effects of motivational interviewing on the progression of patients through the 'stages of change' in smoking cessation in oral and maxillofacial surgery. British Journal of Oral and Maxillofacial Surgery. 2014;52(8):e110. |
| 1. Steliga M, et al. Implementation of a tobacco treatment program within a multidisciplinary thoracic oncology surgery clinic. Journal of Thoracic Oncology. 2013;8(SUPPL. 2):S1295-S1296. |
| 1. Webb A. Smoking cessation strategies at public hospital preadmission clinics in Victoria, NSW and the ACT. Anaesthesia and Intensive Care. 2012;40(5):892. |
| 1. Membrillo MJ, et al. Perioperative tobacco: A simple blind study of the anaesthesiologists' practice. European Journal of Anaesthesiology. 2011;28(SUPPL. 48):74. |
| 1. Akbar M, et al. Surgical trainees and smoking cessation advice - Are we forgetting to use this important resource?. Journal of Vascular Access. 2011;12(1):104. |
| 1. Moller A, et al. Pre-operative nicotine replacement and smoking cessation counselling reduce post-operative complications. Evidence-Based Healthcare. 2002;6(4):190-191. |
| 1. Lee SM, et al. Effectiveness of a perioperative smoking cessation program: an RCT. 2012;59. |
| 1. Wong J, et al. A perioperative smoking cessation intervention with Varenicline vs. Brief advice. 2016;122(5):S271 |

**No barriers or facilitators (n=42):**

| 1. McLaughlin J, et al. Commissioner, clinician, and patient experiences of a pre-surgical health optimisation programme - a qualitative study. BMC Health Services Research. 2021;21(1). |
| --- |
| 1. Bohlin KS, et al. Smoking cessation prior to gynecological surgery-A registry-based randomized trial. Acta Obstetricia et Gynecologica Scandinavica. 2020;99(9):1230-1237. |
| 1. Mustoe MM, et al. Engagement and Effectiveness of a Smoking Cessation Quitline Intervention in a Thoracic Surgery Clinic. JAMA Surgery. 2020;155(9):816-822. |
| 1. Gräsbeck H, et al. Weak smoking cessation awareness in primary health care before surgery: a real-world, retrospective cohort study. Scandinavian Journal of Primary Health Care. 2020;38(1):42-46. |
| 1. Hames MA, et al. Orthopaedic foot and ankle surgeons' approach to elective surgery in the smoking patient population: a survey study. Current Orthopaedic Practice. 2019;30(5):423-428. |
| 1. Warner DO, et al. Adaptive instruction and learner interactivity in online learning: a randomized trial. Advances in Health Sciences Education. 2019;24(3). |
| 1. Akhavan S, et al. Impact of Smoking Cessation Counseling Prior to Total Joint Arthroplasty. Orthopedics. 2017;40(2):e323-e328. |
| 1. Lilley M, et al. Orthopedic Surgeons' Management of Elective Surgery for Patients Who Use Nicotine. Orthopedics. 2017;40(1):e90-e94. |
| 1. Hajjar WM, et al. Behavior, knowledge, and attitude of surgeons and patients toward preoperative smoking cessation. Annals of Thoracic Medicine. 2016;11(2):132-140. |
| 1. Warner DO, et al. Decision Aid for Cigarette Smokers Scheduled for Elective Surgery. Anesthesiology. 2015;123(1):18-28. |
| 1. Musallam KM, et al. Smoking and the risk of mortality and vascular and respiratory events in patients undergoing major surgery. JAMA Surgery. 2013;148(8):755-762. |
| 1. Warner DO, et al. Clinician-delivered intervention to facilitate tobacco quitline use by surgical patients. Anesthesiology. 2011;114(4):847-855. |
| 1. Browning KK, et al. Implementing the Agency for Health Care Policy and Research's smoking cessation guideline in a lung cancer surgery clinic. Oncology Nursing Forum. 2000;27(8):1248-1254. |
| 1. Boe R, et al. Connecting veterans with smoking cessation services in less than 3 minutes. Journal of the American Association of Nurse Practitioners. 2020;33(8):586-590. |
| 1. He Y, et al. Smoking characteristics and readiness-to-quit status among smokers attending preoperative assessment clinic - a prospective cohort study. Risk Management and Healthcare Policy. 2021;14:2483-2490. |
| 1. Wong J, et al. Utilizing patient E-learning in an intervention study on preoperative smoking cessation. Anesthesia and Analgesia. 2018;126(5):1646-1653. |
| 1. Goodney PP, et al. Feasibility and pilot efficacy of a brief smoking cessation intervention delivered by vascular surgeons in the Vascular Physician Offer and Report (VAPOR) Trial. Journal of Vascular Surgery. 2017;65(4):1152-1160. |
| 1. Bader AP, et al. Risk factor modification behaviors of practicing vascular surgeons. Annals of Vascular Surgery. 2017;41:16-17. |
| 1. Saxony J, et al. Evaluation of a smoking cessation service in elective surgery. Journal of Surgical Research. 2017;212:33-41. |
| 1. Marino KA, et al. Operating on Patients Who Smoke: A Survey of Thoracic Surgeons in the United States. Annals of Thoracic Surgery. 2016;102(3):911-916. |
| 1. Zaballos M, et al. Preoperative smoking cessation counseling activities of anesthesiologists: a cross-sectional study. BMC Anesthesiology. 2015;15(1):60. |
| 1. Warner DS, et al. Feasibility of tobacco interventions in anesthesiology practices: A pilot study. Anesthesiology. 2009;110(6):1223-1228. |
| 1. Wolfenden L, et al. Increasing smoking cessation care in a preoperative clinic: A randomized controlled trial. Preventive Medicine. 2005;41(1):284-290. |
| 1. Rohrich RJ, et al. Planning elective operations on patients who smoke: Survey of North American plastic surgeons. Plastic and Reconstructive Surgery. 2002;109(1):350-355. |
| 1. Rozinthe A, et al. Impact of smoking cessation on healing after foot and ankle surgery. Orthopaedics & traumatology, surgery & research. 2022;(101494830):103338. |
| 1. Schaaf D. Smoking Cessation Counseling in the PreAnesthesia Testing Clinic: A Quality Improvement Initiative. Journal of perianesthesia nursing. 2022;9610507. |
| 1. Stonesifer C, et al. Improving smoking cessation referrals among elective surgery clinics through electronic clinical decision support. Tobacco prevention & cessation. 2021;7(101693412):14. |
| 1. Rao BM, et al. Mandatory Nicotine Cessation for Elective Orthopedic Hip Procedures Results in Reduction in Postoperative Nicotine Use. Cureus. 2020;12(12):e12158. |
| 1. Howard R, et al. Impact of a regional smoking cessation intervention for vascular surgery patients. Journal of vascular surgery. 2022;75(1):262-269. |
| 1. Hart A, et al. Smoking Cessation Before and After Total Joint Arthroplasty-An Uphill Battle. The Journal of arthroplasty. 2019;34(7S):S140-S143. |
| 1. Spangler EL, et al. Design and initial enrollment in the Vascular Physicians Offer and Report (VAPOR) trial. Journal of vascular surgery. 2016;63(4):1121-5.e2. |
| 1. Lee SM, et al. Long-term quit rates after a perioperative smoking cessation randomized controlled trial. Anesthesia and analgesia. 2015;120(3):582-587. |
| 1. Lee SM, et al. The effectiveness of a perioperative smoking cessation program: a randomized clinical trial. Anesthesia and analgesia. 2013;117(3):605-613. |
| 1. Hoel AW, et al. Variation in smoking cessation after vascular operations. Journal of vascular surgery. 2013;57(5):1338-4. |
| 1. Kozower BD, et al. A thoracic surgeon-directed tobacco cessation intervention. The Annals of thoracic surgery. 2010;89(3):926-930. |
| 1. Mason DP, et al. Impact of smoking cessation before resection of lung cancer: a Society of Thoracic Surgeons General Thoracic Surgery Database study. The Annals of thoracic surgery. 2009;88(2):362-1. |
| 1. Sillesen H, et al. Organising a nurse-driven PAD rehabilitation clinic within the vascular surgical department: what is required and are treatment goals reached--a prospective study?. European journal of vascular and endovascular surgery. 2007;33(1):26-32. |
| 1. Steinemann S, et al. Impact of education on smoking cessation counseling by surgical residents. American journal of surgery. 2005;189(1):16224. |
| 1. Akoz T, et al. If you continue to smoke, we may have a problem: smoking's effects on plastic surgery. Aesthetic plastic surgery. 2002;26(6):477-82. |
| 1. Hajek P, et al. Brief intervention during hospital admission to help patients to give up smoking after myocardial infarction and bypass surgery: randomised controlled trial. BMJ (Clinical research ed.). 2002;324(7329):32021. |
| 1. Wong J, et al. A Perioperative Smoking Cessation Intervention With Varenicline, Counseling, and Fax Referral to a Telephone Quitline Versus a Brief Intervention: a Randomized Controlled Trial. Anesth Analg. 2017;125(2): 571-579. |
| 1. Smoking Cessation Intervention for Cancer Patients. NCT00575718. <https://clinicaltrials.gov/ct2/show/NCT00575718>   **Non-empirical work (n=7):** |

1. Surgeons help patients quit smoking before surgery. Same-Day Surgery. 2016;40(5):16-16.
2. Arora R, et al. Cardioprotective strategies to improve long-term outcomes following coronary artery bypass surgery. Journal of Cardiac Surgery. 2006;21(2):198-204.
3. Matulewicz RS, et al. Urologist-led smoking cessation: a way forward through implementation science. Translational Andrology and Urology. 2021;10(1):45118.
4. Khullar D, et al. Helping smokers quit around the time of surgery. JAMA - Journal of the American Medical Association. 2013;309(10):993-994.
5. Wolfenden L, et al. Providing comprehensive smoking cessation care to surgical patients: the case for computers. Drug and alcohol review. 2009;28(1):60-65.
6. Rosenthal JS, et al. The responsibility of plastic surgeons to help patients stop smoking [4]. Plastic and Reconstructive Surgery. 2002;109(3):1201-1202.
7. Wright E, et al. Effect of Smoking on Joint Replacement Outcomes: Opportunities for Improvement Through Preoperative Smoking Cessation. Instructional course lectures. 2016;65:509-20.

**Non-English article (n=6):**

1. Pei H, et al. Effect of preoperative smoking intervention on postoperative complications of total hip replacement. Chinese Journal of Evidence-Based Medicine. 2014;14(4):399-403.
2. Shaw H, et al. Smoking addiction and patients' motivation to quit it among attenders of the Saldent 2006 conference. Przeglad lekarski. 2006;63(10):1075-1077.
3. Shimizu Y, et al. [Smoking cessation program run by anesthesiologists in a preoperative clinic]. Masui. The Japanese journal of anesthesiology. 2013;62(11):1372-4.
4. Kato M, et al. [Survey of the preoperative smoking cessation in patients for elective surgery in a university hospital]. Masui. The Japanese journal of anesthesiology. 2013;62(8):1013-6.
5. Derlon V, et al. [Management of smoking in the perioperative period: survey of practices amongst anaesthetists in Lorraine]. Annales francaises d'anesthesie et de reanimation. 2013;32(2):89-93.
6. Zhang L, et al. [Smoking cessation rate in cardiovascular patients after percutaneous coronary intervention]. Zhonghua yi xue za zhi. 2011;91(12):815-8.

**Reviews and meta-analyses (n=4):**

1. Stokes SM, et al. Optimizing health before elective thoracic surgery: Systematic review of modifiable risk factors and opportunities for health services research. Journal of Thoracic Disease. 2019;11(Supplement4):S537-S554.
2. Thomsen T, et al. Interventions for preoperative smoking cessation. Cochrane Database of Systematic Reviews. 2014;(3).
3. Cahill K, et al. Pharmacological interventions for smoking cessation: an overview and network meta-analysis. Cochrane Database of Systematic Reviews. 2013;(5).
4. Cahill K, et al. Nicotine receptor partial agonists for smoking cessation. Cochrane Database of Systematic Reviews. 2016;(5).

**Wrong population (n=7):**

1. Luxton NA, et al. Use of electronic cigarettes in the perioperative period: A mixed-method study exploring perceptions of cardiothoracic patients in Australia. Tobacco Induced Diseases. 2018;16:44937.
2. Tønnesen H, et al. Risk reduction before surgery. The role of the primary care provider in preoperative smoking and alcohol cessation. BMC Health Services Research. 2010;10:121-121.
3. Easton A, et al. Non-primary care physicians and smoking cessation counseling: Women Physicians' Health Study. Women & Health. 2001;34(4):15-29.
4. Riley H, et al. Smoking cessation after hospitalization for myocardial infarction or cardiac surgery: Assessing patient interest, confidence, and physician prescribing practices. Clinical Cardiology. 2019;42(12):1189-1194.
5. Bader AP, et al. Risk Factor Modification Behaviors of Practicing Vascular Surgeons. Annals of Vascular Surgery. 2018;46:90-96.
6. Dulger S, et al. Analysis of the Role of Physicians in the Cessation of Cigarette Smoking Based on Medical Specialization. Clinics (Sao Paulo, Brazil). 2018;73:e347.

Shi Y, et al. Surgery as a teachable moment for smoking cessation. Anesthesiology. 2010;112(1):102-107.
