# Supplementary material for: Barriers and facilitators to perioperative smoking cessation: A scoping review
Source: PLoS One. 2024 Jun 11;19(6):e0298233. doi: 10.1371/journal.pone.0298233 (PMC11166293; doi:10.1371/journal.pone.0298233)
Supplement: Attachment — Submitted filename: Response to Reviewers.docx [file pone.0298233.s006.docx]

**RESPONSE TO EDITOR’S AND REVIEWER’S COMMENTS**

We thank the reviewers for taking the time to provide us with their thorough and constructive comments and suggestions on our manuscript.

**Journal Requirements:**

| **Journal Requirements** | **Authors’ Response** |
| --- | --- |
| Please ensure that your manuscript meets PLOS ONE's style requirements, including those for file naming. | We have done so. |
| State source of funding | We acknowledge that we selected the wrong option while submitting the manuscript on the Journal online submission platform. We received no funding for this work, so we have corrected to “The author(s) received no specific funding for this work”. |
| We note that you have stated that you will provide repository information for your data at acceptance. Should your manuscript be accepted for publication, we will hold it until you provide the relevant accession numbers or DOIs necessary to access your data. | Our data is freely available at https://osf.io/z3pyd/?view_only=c621853d3da0494082da159907058be6 |
| Please amend either the title on the online submission form (via Edit Submission) or the title in the manuscript so that they are identical. | We have now done so. |
| We note that this manuscript is a systematic review or meta-analysis; our author guidelines therefore require that you use PRISMA guidance to help improve reporting quality of this type of study. Please upload copies of the completed PRISMA checklist as Supporting Information with a file name “PRISMA checklist”. | This is now uploaded separately. |
| Please review your reference list to ensure that it is complete and correct. | This has been reviewed and is complete and correct. |

**Reviewer #1:**

| **Reviewer Comments** | **Authors’ Response** |
| --- | --- |
| The Short title applies to a wider scope | We thank the reviewer for bringing this to our attention. We have modified our short title so that it is clear that these barriers and facilitators apply to the perioperative period.  Our short title now reads: “Barriers and facilitators to perioperative smoking cessation”. |
| Please clarify what informed the choice of the search engines | Our choice of bibliographic databases was informed by our consultation with a health sciences librarian. A combination of large (MEDLINE, Embase, CENTRAL), and topic focused (CINAHL, PsycINFO) databases were chosen to optimize the retrieval of relevant records. |
| Background- the figure 2.4%, not clear if it applies to the whole population or only to smokers | We thank the reviewer for this comment. To clarify, this 2.4% refers to patients who were smoking in the 4 weeks prior to their surgery.  This is outlined in lines 62 to 66. “In an analysis from the VISION study, a large prospective cohort study of a representative sample of 40,004 patients aged ≥45 years who underwent major noncardiac surgery across 28 centers in 14 countries, we found that, among the 5480 (13.7%) who were smoking in the 4 weeks preceding their surgery, only 2.4% received any smoking cessation pharmacotherapies before surgery and 6.5% after surgery”. |
| Provide key or footnote in your diagrams and tables | Thank you. We completely agree that key notes and footnotes are essential to clarify information in tables. For tables where acronyms have been used (e.g., Table 1), we have included foot notes clarifying these acronyms. Our remaining tables do not have acronyms. |

**Reviewer #2:**

Thanks for submitting this manuscript. I have multiple points to the authors to consider or to clarify on the manuscript:

| **Reviewer Comments** | **Authors’ Response** |
| --- | --- |
| Please state the qualifications of the reviewers including the third one. Line 122 and 126. | Although it is not common practice to explicitly outline the qualifications of systematic review authors, it is important that those conducting the systematic review have the appropriate methodological expertise.  All reviewers are individuals with expertise in Health Research Methodology and are academic staff at McMaster University (the second and third are Master’s students) and have extensive experience conducting systematic reviews. The third reviewer has previous experience with systematic reviews and was guided throughout this process. Furthermore, we ensured that all authors participating in the screening process of the review fully understood our inclusion and exclusion criteria by pre-piloting a set of 10 references prior to screening of the remaining articles.  All steps regarding search, screening, data extraction and quality assessment were done independently and in duplicate to ensure compliance with systematic review methods. |
| Could you state whether the selected studies were theory-base or not. Table 1. | Of all the included studies, only one was theory based. The authors cited that they were guided by “social cognitive theory and the conflict theory of decision making”. (Reference: McDonnell KK, Bullock LF, Kozower BD, Hollen PJ, Heath J, Rovnyak V. A decision aid to improve smoking abstinence for families facing cancer. Oncol Nurs Forum. 2014;41(6):649–58).  We have now included this in the manuscript. |
| Please review line 260 for miss spelling reported. | We thank the reviewer for bringing this to our attention. This spelling mistake has been corrected. |
| Please review line 382 for clarity. | Thank you for bringing this sentence to our attention. We have reworded these sentences to be clearer.  They now read: “In a busy perioperative service in a Canadian hospital, this type of care integration increased the rate of identifying smokers; pre-implementation only 8.6% of patients were asked about smoking whereas, post-implementation, this rose to 86.0%. Consequently, the rate of providing an initial treatment intervention to smokers increased from 0% to 67.5% but there was no data on whether this improved smoking cessation rates”. |
| I suggest splitting table 2 to two tables. One for health care providers and one for patients only for easy reading. | Thank you for your thoughtful feedback and suggestion to separate the current table into two distinct ones, each focusing on the perspectives of healthcare providers and patients respectively.  Upon reflection and thorough consideration of your recommendation and considering that the contents of the primary column would remain identical across both proposed tables, in our view, this could potentially lead to unnecessary repetition and could detract from the ease of comparison and comprehension for the readers.  The decision to present the perspectives of both healthcare providers and patients within a single table was driven by a desire to facilitate direct comparison and juxtaposition of these separate critical viewpoints. We believe that the current format allows for an at-a-glance understanding of healthcare providers and patients’ perspectives, enhancing readability, and reducing the need for cross-referencing between multiple tables.  We certainly appreciate your concern and understand the rationale behind the suggestion. However, in the context of maintaining a streamlined presentation and fostering a more intuitive comparison of viewpoints, we propose to retain the current table format. |
