# Supplementary material for: Barriers and facilitators to perioperative smoking cessation: A scoping review
Source: PLoS One. 2024 Jun 11;19(6):e0298233. doi: 10.1371/journal.pone.0298233 (PMC11166293; doi:10.1371/journal.pone.0298233)
Supplement: Attachment — Submitted filename: RESPONSE TO REVIEWER.docx [file pone.0298233.s007.docx]

**RESPONSE TO REVIEWER’S COMMENTS**

We sincerely thank all the reviewers and especially reviewer 3 for taking the time to provide us with their thorough, insightful, and constructive comments, and suggestions on our manuscript. We have provided a response to all the reviewer’s comments and revised the manuscript accordingly. To aid in your review, any new additions we made to the manuscript are highlighted in red here below with manuscript location referring to the line and page number in the “clean version” of the manuscript.

We have re-submitted 2 versions of the manuscript; one with “tracked changes” and a “clean” version. We hope that the reviewer finds our responses satisfactory.

**Journal Requirements:**

Please review your reference list to ensure that it is complete and correct. If you have cited papers that have been retracted, please include the rationale for doing so in the manuscript text or remove these references and replace them with relevant current references. Any changes to the reference list should be mentioned in the rebuttal letter that accompanies your revised manuscript. If you need to cite a retracted article, indicate the article’s retracted status in the References list and also include a citation and full reference for the retraction notice.

**Response**: We have reviewed the reference list, and it is complete and correct.

**Review Comments to the Author from Reviewer #3**

1. Introduction, line 68 – I think you mean systems-led behavior change or system-level support to lead to behavior change?

**Response**: Thank you, we have edited to read “systems-led behavior change”

**Manuscript location**: line 68, page 3.

2. Section 2.1, add appendix reference next to mesh terms so people know where they can find it.

**Response**: We have now re-written this to read “We consulted a health sciences librarian with expertise in quantitative and qualitative methods, and developed a search strategy for MEDLINE, Embase, Cochrane Central Register of Controlled Trials (CENTRAL), CINAHL, and PsycINFO databases using keywords and MeSH terms (Appendix II)”.

**Manuscript location**: line 107, page 5.

3. Section 2.2 should be cleaned up; you are referring to eligibility of all the studies but it is a bit confusing with the participants and semi-colon. Maybe start broad e.g., criteria as it related to all studies and then specify that studies could include your specified population.

**Response:** we have removed the “participants:” for clarity.

You also seem to be missing more specification for your exclusion criteria, a lot of the articles you exclude on in the PRISMA are not specified criteria in this section.

**Response:** These were omitted for brevity but we have now included “We included studies published in 2000 or later, in English,….. “We excluded studies not involving our population of interest, reviews, or editorials, and theses and conference abstracts that were not published in full.”

**Manuscript location**: lines 115 and 119, page 5.

These sentences are found in the section above eligibility criteria but are not in the appropriate section: The search was restricted to English language articles published in 2000 or later. AND We screened systematic reviews for individual studies that met the eligibility criteria.

**Response**: This sentence reflects our search strategy, that is why we left it in the “search strategy” section. We have now however edited the eligibility section to include studies published in 2000 or later and in English. Even though we excluded systematic reviews, we screened them for individual studies (included in the reviews) that met our criteria. We have edited to “We also screened systematic reviews for individual studies that met the eligibility criteria”. We hope that this provides clarity.

**Manuscript location**: line 110, page 5.

4. Could the authors include a citation for the approach to quality reporting (section 2.5) starting on line 138-9? Is this standard practice, some justification for this approach is necessary.

**Response**: With the Mixed Methods Appraisal Tool (MMAT) cited in line 138, there is no clearly defined scoring system. What is recommended is to clearly describe how the results of the appraisal were interpreted and used in the review. This is explained in the frequently asked questions document that accompanies the MMAT as can be found here: <http://mixedmethodsappraisaltoolpublic.pbworks.com/w/file/fetch/140056890/Reporting%20the%20results%20of%20the%20MMAT.pdf>

We have added this: “There is no clearly defined scoring system with this tool, and we chose to report the final reporting quality as the percentage of the number of reporting items divided by the number of total items evaluated.”

**Manuscript location**: line 138-140, page 6.

5. I find this sentence really confusing in the data synthesis section: From quantitative studies, we summarized the proportion of participants who report negative or permissive attitudes towards smoking and provide smoking cessation support/interventions as numbers and percentages. I don’t think a reader could replicate this, could you be clearer in how someone would reproduce this approach.

**Response**: What we meant by this is that we provided counts (number and percent) of study participants who report negative or permissive attitudes towards smoking (e.g., line 339 “In a survey of 542 anesthesiologists and 521 surgeons, physicians who smoked were less likely to agree that the perioperative period was a good time to help patients stop smoking (15% of smokers vs 36% and of non-smokers) and counts (number and percent) of study participants who provide smoking cessation support/interventions (e.g., line 225 “In a study involving 92 perioperative healthcare providers, those who believed that counseling patients would increase quit rates were significantly more likely to advise patients to quit, with 52.1% of believers taking time to counsel their patients compared to 0.0% of non-believers).

However, considering that it should be easier for readers, we have rephrased that section to “We extracted data on what participants considered as barriers and facilitators to smoking cessation and we present the number of participants who express these within each study, as numbers and percentages.”

**Manuscript location**: lines 153-155, page 7.

6. You use HCP on page 7 line 173 for the first time and don’t define it.

**Response**: We have now defined it in line 173, to “health care providers (HCPs).”

**Manuscript location**: line 173, page 8.

7. Results, the authors don’t really address that family members are included in some of these studies as are there perspectives, should this not be added in some way beyond Table 1, or if the authors choose not to report it, state that and why? It looks like it is only brought up once in the results.

**Response**: We agree with the reviewer that the family member’s perspective could be important but, in this review, we did not specifically search for studies looking at this issue from the family perspective. Our study focuses on patient and healthcare provider perspective. Two studies included patients and their family members and our findings from this indicate that patients feel obtaining advise from family, and interventions that involve their family are helpful which we show on table 2.

8. It seems like the authors report on “other” in Table 2, is the family members? Could you better define this in the table – what is this subgroup comprised of?

**Response**: Here we mean “other” includes residency program directors (could be different professionals). We have added a footnote to table 2.

9. Could the authors explain how they differentiate between capabilities vs. motivation? Are these articles actually talking about self-efficacy overall or is motivation salient here, if so, should the table be updated?

**Response**: We agree that motivation is likely a salient factor here as it is with many aspects of behavior change. However, we specifically mapped our identified barriers and facilitators to the domains of the theoretical domain framework (TDF) and ‘belief about capabilities’ is one of the 14 domains of the TDF. Here, capabilities refer to perceived competence or self-confidence/professional confidence which we mapped unto this domain. Motivation was removed from the latest TDF version and replaced by “intentions” and “goals”. Constructs under this would include for example, stability of intentions, stages of change, and action planning.^1^

10. Aren’t these two items in table 2, category 6, not knowledge issues? Misperceptions? - Barrier: indication for surgery not related to smoking

Barrier: underestimation of the effect of smoking cessation on postop complications

**Response**: It is a judgement call when mapping identified themes onto the various TDF domains, and for this reason we did this in duplicate. One could make a case that they are indeed knowledge issues, but we felt they more closely represent a belief about the consequences of smoking. For example, in Taniguchi et al,^2^ the subjects (nurses) ***felt*** *that the need for tobacco use intervention was significantly higher in patients with tobacco-related cancers than in those with non- tobacco-related cancers.* Similarly in Warner et al,^3^ surgeons and anesthesiologists were queried on their beliefs about benefits of short term <30 days abstinence from smoking and *only 52% of anesthesiologists and 70% of surgeons* ***believed*** *it was useful to reduce postoperative complications.*

11. Table 2, category 8, is lack of intentions the right word here? The way it is described in the table doesn’t seem like it’s a lack of intention on the part of the providers but a lack of belief in the outcome from the providers perspective, also by stage of change do you mean perceived readiness to change?

**Response**: You are correct, and we have changed to perceived readiness to change, and we have corrected in the table.

**Manuscript location**: table 2, number 8.

By intentions here we refer to “a conscious decision to perform a behaviour or a resolve to act in a certain way.”^1^ In Yao et al, “only 27% of the male surgeons always advised their patients to quit smoking. Among those respondents who never or seldom advised their patients to quit, the main reason given was *the belief that ‘‘they could not influence a patient”*. Our interpretation of this is that their lack of intent to provide smoking cessation advise is driven by their intrinsic belief that they cannot influence behaviour. This would have fallen under “motivations” domain in the earlier iteration of the TDF (version 1).

12. Also, a general comment is you do have a category that is decision processes, it seems to me that anything related to stages of change or motivation to change should go here - Suggest revising.

**Response**: We understand the reviewers good point. However, since our goal is to map the identified barriers and facilitators unto the 14 TDF domains which are pre-defined, we are constrained to do so within the framework. We understand the concern with this and have now included this important factor as a study limitation on page 24. In the TDF, anything related to stages of change or motivation to change would go to the “intention domain” or “goals” domain. Whereas the definition for Memory, attention and decision processes is “The ability to retain information, focus selectively on aspects of the environment and choose between two or more alternatives”.^1^

13. Could you walk me through why pessimism fits in the optimism theme? It seems like the opposite.

**Response**: In the TDF, “Optimism, Pessimism, Unrealistic optimism and Identity” are constructs within the Optimism domain.^1^

14. The authors need to specifically cite the studies that this relates to in the following sentence on Page 15 - Lack of hospital support regarding accessibility and availability of quit kits [cite], Quitline referral materials [cite], nicotine replacement therapy [cite], and lack of integration of care between all professionals [cite] and hospital environments [cite] involved in the patient's care were some of the identified barriers within this theme [23].

**Response**: This particular sentence was from one study. In the cited reference,^4^ “Inadequate hospital support included: the lack of accessibility and availability in hospital pharmacies to provide *NRT*, and the absence of SC care documentation between professionals and hospital environments throughout the patient’s perioperative period. Scarce resources such as *Quit kits* and *Quitline referral pads* created a feeling of administrative disinterest. The desire to link a patient to specialist face-to- face help was limited by the existence of only two specialist clinics (with limited availability) for the six hospitals.”

15. Page 15, suggest you add what country this is in as there is a lot of variation in this study: In one study of 2,740 surgeons, 215 40.2% strongly agreed with the statement “reimbursement issues prevent me from providing 216 tobacco-use cessation services”?

**Response**: We have edited to “In one study of 2,740 surgeons in the United States of America (USA),…..”.

**Manuscript location**: Line 214, page 15.

16. Under the knowledge heading on page 16, the citations should not all be piled at the end but linked to the specific points in the sentence, please revise.

**Response:** This has been revised as requested to “inadequate knowledge of the five steps for smoking intervention known as the 5A's (Ask, Advise, Assess, Assist, and Arrange treatment) and other counseling techniques [19,20,22,23,39], and limited knowledge of NRT efficacy and how to help smokers [20, 55].”

**Manuscript location**: Lines 232 and 233, page 16.

17. Under the knowledge heading, was this pattern globally providers had a misconception? Was it restricted to certain regions? This would be important specification to add.

**Response**: We have now added the specific locations (USA and United Kingdom) in the text.

**Manuscript location:** Lines 235 and 238, page 16.

18. Skills section, first sentence could you move the citations to the training or awareness section so the reader can know what article links to what statement?

**Response**: We have edited to “Across 5 studies, surgeons, anesthesiologists, and nurses identified that training on smoking cessation [43, 51, 54, 58] and awareness of smoking cessation guidelines [22, 43, 51, 58] were important facilitators.”

**Manuscript location:** Lines 324 and 325, page 20.

19. Intentions heading, you have readiness to change in the table, but you don’t comment on it in this section.

**Response**: We have edited to “Providers’ beliefs about their inability to influence patients and their perceived patients’ lack of readiness to quit, were barriers in this domain.”

**Manuscript location:** Line 329, page 20.

20. For the key findings could you also numerically add the number of studies that outline these findings for each point? e.g., barriers (n) vs facilitators (n)

**Response**: We have edited to: “Our key findings are i.) we identified more barriers (23) than facilitators (13) to perioperative smoking cessation; ii.) the most frequent barriers were related to lack of time (number of studies n=13), limited knowledge of smoking cessation interventions among providers (n=10), and belief that patients are nervous about discussing smoking cessation before surgery (n=7); iii.) key facilitators included leveraging surgery itself as a motivator (n=7) to quit smoking and providing smoking cessation advice by physicians (n=5).”

**Manuscript location:** Lines 61-367, pages 21-22.

21. Line 372- discussion is an important point, however, given the breadth of studies this review covers, it is a little too broad of a comment. The authors should consider how this comment relates to different health care systems which articles in this review cover, I would imagine some countries do have very different reimbursements or even some specialities might have more integration than others? I would suggest the authors spend some time looking at the differences and revise this section accordingly, so it isn’t just western based recommendations or at least accounts for different healthcare systems.

**Response**: We agree with the reviewer however given word count constraints, we have added “Except for one study [47], all were conducted in high and upper-middle-income countries. Since reimbursement systems differ across healthcare settings, any system-level changes must be tailored to the specific context.”

**Manuscript location**: Lines 377-379, page 22.

22. Same reflection to line 391 – discussion, is the Cochrane review restricted to certain regions or healthcare systems? Specify

**Response**: As per the Cochrane report “Eleven of the 17 studies were conducted in the USA, one in Canada (Wilson 1988), one in Taiwan (Wang 1994), one in Scotland (Sinclair 1998), one in the United Kingdom (Lennox 1998), one in Switzerland (Cornuz 2002) and one in Germany (Twardella 2007).”^5^

Due to word count constraints, we have not gone into detail about this, but we cite the reference for readers and include: “A Cochrane review of 17 RCTs from mostly high-income countries demonstrated that training health professionals to provide smoking cessation interventions”.

**Manuscript location**: Line 396, page 23.

23. The first part of this sentence needs a citation: There is compelling evidence that patients who smoke want to quit, [cite] and we found in this review that surgery itself is a facilitator for smoking cessation [65,66].

**Response**: We have revised to remove the word ‘compelling’ and the reference has been added: There is compelling evidence that patients who smoke want to quit [7].

**Manuscript location**: Line 408, page 23.

24. Is this assertion true for this part of the sentence on line 411, discussion? - helping patients quit smoking for surgery has the potential for long-term abstinence? – could you cite

**Response**: We have provided a reference for this statement: Moreover, beyond reducing complications, helping patients quit smoking for surgery has the potential for long-term abstinence [71]

**Manuscript location**: Line 416, page 24.

25. Limitations – given that you used a theoretical framework you tried to fit your themes into this framework rather than generate them inductively, you may want to mention this, I also think the authors needs to comment on generalizability as some of the articles were very small samples vs others and that there was large heterogeneity across the articles. Also missing authors bias, there seems to be a high level of subjectivity about the quality of the articles and the MMAT assessment, it seems like this should be acknowledged.

**Response**: We have added the following: “Limitations of this review include firstly, we only including articles published in English which were heterogenous and of varied sample sizes, potentially limiting its generalizability to other non-English speaking settings and with different sociocultural and environmental contexts. Secondly, we were unable to quantify whether the identified barriers and facilitators were considered trivial, moderate, or large as this was not assessed in the individual studies. Thirdly, our theme choices were guided by the TDF rather than by strictly inductive analysis. This might have led us to miss unique or unexpected themes that didn't fit the established TDF domains. Lastly, the subjective nature of our quality assessment with the MMAT scoring, might have introduced some author bias.”

**Manuscript location**: Lines 432-440, pages 24-25.

26. Figure 1 prisma, you have *’s that don’t link with anything, could you add footnotes for these symbols and make sure you include why records were excluded in the first part of the diagram too, not just the last.

**Response:** We have removed the ** from the diagram.

In the title and abstract screening stage (which we did on Covidence), we removed a large number of articles that were irrelevant to our review and it is usually not possible on Covidence to record reasons other than that they were irrelevant. (<https://support.covidence.org/help/does-covidence-record-exclusion-reasons-at-the-title-and-abstract-screening-stage>).

Exclusion reasons are selected at the full text stage, and we have included that in the diagram with additional details now added to the supporting information document as appendix III in case the reviewer and/or readers are interested.

**Manuscript location**: supporting information, appendix III.

27. Comment on supplemental materials – the formatting looks a bit messy for the search criteria - extra letters etc. like it was just thrown together, could you clean these up or make the tables consistent in some way?

**Response**: We have tabulated and reformatted the text for all the search strategies. Please note that the text is imported directly from the search engines to ensure accuracy.

28. Table 1 looks ok to me.

**Response**: We thank the reviewer for their comment.

**References**

1.	Atkins L, Francis J, Islam R, O’Connor D, Patey A, Ivers N, et al. A guide to using the Theoretical Domains Framework of behaviour change to investigate implementation problems. Implementation Science. 2017;12(1):77. Available from: https://doi.org/10.1186/s13012-017-0605-9

2.	Taniguchi C, Hibino F, Kawaguchi E, Maruguchi M, Tokunaga N, Saka H, et al. Perceptions and practices of Japanese nurses regarding tobacco intervention for cancer patients. J Epidemiol. 2011;21(5):391–7.

3.	Warner DO, Sarr MG, Offord KP, Dale LC. Anesthesiologists, general surgeons, and tobacco interventions in the perioperative period. Anesth Analg. 2004;99(6):1766–73.

4.	Luxton NA, MacKenzie R, Shih P. Smoking Cessation Care in Cardiothoracic Surgery: A Qualitative Study Exploring the Views of Australian Clinicians. Heart Lung Circ. 2019;28(8):1246–52. Available from: https://doi.org/10.1016/j.hlc.2018.04.293

5.	Carson K V, Verbiest MEA, Crone MR, Brinn MP, Esterman AJ, Assendelft WJJ, et al. Training health professionals in smoking cessation. Cochrane Database Syst Rev. 2012;(5):CD000214. doi: 10.1002/14651858.CD000214
