# Supplementary material for: Barriers and facilitators to perioperative smoking cessation: A scoping review
Source: PLoS One. 2024 Jun 11;19(6):e0298233. doi: 10.1371/journal.pone.0298233 (PMC11166293; doi:10.1371/journal.pone.0298233)
Supplement: Attachment — Submitted filename: Response to Reviewers.docx [file pone.0298233.s008.docx]

**RESPONSE TO REVIEWER’S COMMENTS**

We sincerely thank all the reviewers and especially reviewer 4 for taking the time to provide us with their thorough, insightful, and constructive comments, and suggestions on our manuscript. We have provided a response to all the reviewer’s comments and revised the manuscript accordingly. To aid in your review, any new additions we made to the manuscript are highlighted in red here below with manuscript location referring to the line and page number in the “clean version” of the manuscript.

We have re-submitted 2 versions of the manuscript; one with “tracked changes” and a “clean” version. We hope that the reviewer finds our responses satisfactory.

**Journal Requirements:**

Please review your reference list to ensure that it is complete and correct. If you have cited papers that have been retracted, please include the rationale for doing so in the manuscript text or remove these references and replace them with relevant current references. Any changes to the reference list should be mentioned in the rebuttal letter that accompanies your revised manuscript. If you need to cite a retracted article, indicate the article’s retracted status in the References list and also include a citation and full reference for the retraction notice.

**Response**: We have reviewed the reference list, and it is complete and correct.

**Review Comments to the Author from Reviewer #4**

1. In general, the background has a bit too much detail on prior work (particularly lines 80-88) - would keep intro more succinct and add this level of detail to the discussion.

**Response**: We appreciate this feedback and have significantly shortened the highlighted section to maintain some background information for readers but more concisely. The text now reads “Previous studies have utilized the TDF framework to identify key factors affecting smoking cessation in various groups. Campbell et al. found that existing smoking cessation methods for pregnant women don't sufficiently address social network influences, a major barrier [26]. Huddlestone et al. identified time constraints as the main obstacle for smoking cessation in mental health settings, with effective support materials being the most helpful enabler [27].”

**Manuscript location**: lines 79-83, page 4.

2. Commas are overused in several places in the manuscript

**Response**: Thank you for your comment regarding comma usage in our manuscript. We would like to note that there are various styles of comma usage, and for this manuscript, we have adopted the Oxford comma style for consistency.

3. "Hand-searched" is an odd term - were the hands actually used to search? Or are you indicating that the references were individually reviewed by the authors?

**Response**: To clarify, we manually searched the reference lists of relevant reviews to identify articles for screening, a method commonly employed in systematic reviews and endorsed by the PRISMA guidelines.^1^ This approach is also exemplified in recent publications in the PLOS One Journal.^2,3^ Nonetheless, in deference to your suggestion, we have revised the term to “manually-searched”.

**Manuscript location**: line 104, page 5.

4. It is generally frowned upon to refer to patients by only the noun "smokers" (line 114) instead of "patients who were active smokers" or "patients who smoke".

**Response**: We have edited this to “adults who smoke”.

**Manuscript location**: line 109, page 5.

5. Its confusing to the readers how RCTs would address barriers/facilitators in the methods section, though clarified later when it is stated that they had additional qualitative analysis performed.

**Response**: We thank the reviewer for this comment.

6. were the 10 pilot screening references chosen at random? Or were they the first (and likely most relevant) 10?

**Response**: They were randomly chosen. We have clarified in the manuscript by adding “randomly chosen”

**Manuscript location**: Line 117, page 5.

7. Please provide more detail about Covidence (company, location is usually appropriate when referring to a software in a scientific manuscript)

**Response**: We have added detail: “Covidence systematic review software, Veritas Health Innovation, Melbourne, Australia. Available at [www.covidence.org](http://www.covidence.org)”.

**Manuscript location**: Lines 120-121, page 5.

8. Do not repeat data that is in tables in text (including location of studies). This will help with the referenced word limits in response to other reviewer comments.

**Response**: We have now deleted details about study location from the text.

9. Table 2 - "physicians and surgeons should provide advice to quit smoking..." how is a "should" statement a facilitator? Do you mean that if they BOTH did it was a facilitator? It's confusing.

**Response**: We have now clarified by editing to “and/or” in Table 2.

10. How are perceived health benefits a barrier - does that mean "lack of" perceived benefits?

**Response**: We have now clarified by editing to “perceived health benefits of smoking”. This refers to the finding from one study included in the review that patients believed smoking could be beneficial (Line 236, page 16 in the results section under the subheading “Knowledge”).

11. Knowledge "on" is used often - do they mean knowledge of?

**Response**: We thank the reviewer for their careful review. These have now been corrected to “knowledge of”.

12. Why is 8: lack of intention - "providers' belief.." not in the belief section?

**Response**: We appreciate this comment and acknowledge that there is some subjectivity inherent in conducting this review using the Theoretical Domains Framework (TDF).^4^ This is precisely why we ensured that the coding was done in duplicate. According to the TDF, 'Intentions' are defined as a conscious decision to perform a behavior or a resolve to act in a certain way. In contrast, the 'Belief about Capabilities' domain considers factors like self-efficacy. In our study, within the Intentions domain, we determined that it was not the providers' lack of self-efficacy that prevented them from offering support. Rather, they chose not to provide smoking cessation advice because they believed it would not influence the patients to change. As stated in the main text, “Surgeons who did not advise patients to quit smoking cited their inability to influence them as the primary reason (39.4%).” We hope the reviewer can understand our perspective. In addition, we discuss this subjectivity as a limitation of this study.

13. In the discussion authors state (line 233) that there is a "misconception" that interventions were not efficacious - in fact recent systematic reviews have supported this conception.

**Response**: We have now corrected this and state “Providers also believed…”

**Manuscript location:** line 228, page 16.

14. Must of the discussion is biased in statements that interventions help but not supported by the data - for example line 301-302 reports on beliefs but lacks robust data as to actual outcomes.

**Response**: We appreciate this comment and acknowledge the reviewer's concern regarding the lack of robust data. However, we wish to clarify that the sections in question reflect the findings from studies included in our scoping review, rather than our own viewpoints.

15. Similarly in lines 388-390.

**Response**: We thank the reviewer and agree that the study cited in lines 388-390 buttresses the point above which again is a result from an included study in this scoping review and not our viewpoint.

16. Overall discussion rehashes much of the results without substantial additional information - would be helpful to make more concise and summarize what of your results is new/additive to the literature in each area.

**Response**: We have deleted sections from the discussion (as seen on the tracked changes version). We have now added: “What is new: our study is the first theory-informed scoping review examining barriers and facilitators to smoking cessation in the perioperative setting. Key finding include: i.) more barriers (23) than facilitators (13) to perioperative smoking cessation were identified; ii.) primary barriers encompass lack of time (number of studies n=13), limited knowledge among providers (n=10), and belief about patient apprehension about pre-surgery cessation discussions (n=7); iii.) significant facilitators are using surgery as a motivation to quit (n=7) and physician-provided cessation advice(n=5). Notably, the link between these factors and cessation success remains unexplored. Our findings indicate a need for a comprehensive approach to implement effective perioperative smoking cessation strategies, addressing multiple identified barriers across several domains.”

Manuscript location: First paragraph of discussion section, page 21.

**References**

1.	Rethlefsen ML, Kirtley S, Waffenschmidt S, Ayala AP, Moher D, Page MJ, et al. PRISMA-S: an extension to the PRISMA Statement for Reporting Literature Searches in Systematic Reviews. Syst Rev. 2021;10(1):1–19.

2.	Kilpatrick K, Tchouaket E, Savard I, Chouinard MC, Bouabdillah N, Provost-Bazinet B, et al. Identifying indicators sensitive to primary healthcare nurse practitioner practice: A review of systematic reviews. PLoS One. 2023;18(9):e0290977.

3.	Vinadé Chagas ME, Rodrigues Moleda Constant HM, Cristina Jacovas V, Castro da Rocha J, Galves Crivella Steimetz C, Cotta Matte MC, et al. The use of telemedicine in the PICU: A systematic review and meta-analysis. PLoS One. 2021;16(5):e0252409.

4.	Atkins L, Francis J, Islam R, O’Connor D, Patey A, Ivers N, et al. A guide to using the Theoretical Domains Framework of behaviour change to investigate implementation problems. Implementation Science [Internet]. 2017;12(1):77.
